# Neurog2 directly converts astrocytes into functional neurons in midbrain and spinal cord

**DOI:** 10.1038/s41419-021-03498-x

**Published:** 2021-03-01

**Authors:** Fei Liu, Yijie Zhang, Fuliang Chen, Jiacheng Yuan, Sanlan Li, Sue Han, Dengyu Lu, Junlan Geng, Zhiping Rao, Li Sun, Jianhua Xu, Yuhan Shi, Xiaojing Wang, Yueguang Liu

**Affiliations:** 1 Anhui Clinical and Preclinical Key Laboratory of Respiratory Disease; Molecular Diagnosis Center, Department of Pulmonary and Critical Care Medicine, The First Affiliated Hospital of Bengbu Medical College, Bengbu, Anhui 233000 China; 2 University of Chinese Academy of Sciences, Beijing, 100049 China; 3 Department of Immunology, School of Laboratory Medicine, Anhui Province Key Laboratory of Immunology in Chronic Diseases, Bengbu Medical College, Bengbu, Anhui 233030 China; 4 Department of Radiation Oncology, The First Affiliated Hospital of Bengbu Medical College, Bengbu, 233004 China; 5 Department of Neurology, Jiading District Central Hospital Affiliated to Shanghai University of Medicine & Health Sciences, Shanghai, 201800 China

**Keywords:** Neuronal development, Adult neurogenesis

## Abstract

Conversion of astrocytes into neurons in vivo offers an alternative therapeutic approach for neuronal loss after injury or disease. However, not only the efficiency of the conversion of astrocytes into functional neurons by single Neurog2, but also the conundrum that whether Neurog2-induced neuronal cells (Neurog2-iNs) are further functionally integrated into existing matured neural circuits remains unknown. Here, we adopted the AAV(2/8) delivery system to overexpress single factor Neurog2 into astrocytes and found that the majority of astrocytes were successfully converted into neuronal cells in multiple brain regions, including the midbrain and spinal cord. In the midbrain, Neurog2-induced neuronal cells (Neurog2-iNs) exhibit neuronal morphology, mature electrophysiological properties, glutamatergic identity (about 60%), and synapse-like configuration local circuits. In the spinal cord, astrocytes from both the intact and lesioned sources could be converted into functional neurons with ectopic expression of Neurog2 alone. Notably, further evidence from our study also proves that Neurog2-iNs in the intact spinal cord are capable of responding to diverse afferent inputs from dorsal root ganglion (DRG). Together, this study does not merely demonstrate the feasibility of Neurog2 for efficient in vivo reprogramming, it gives an indication for the Neurog2-iNs as a functional and potential factor in cell-replacement therapy.

## Introduction

Dysfunction of the central nervous system in traumatic injury and neurodegenerative diseases is associated with neuronal cell loss. However, adult neurogenesis primarily occurs in a few specific areas of the brain, such as the subventribular zone (SVZ) lining the lateral ventricles, as well as the subgranular zone within the dendate gyrus (DG) of the hippocampus, whose primary function points to the process of brain plasticity rather than brain repair^1,2^. Several strategies have been employed for the post-lesion rejuvenation of neuronal functions comprising cell rescue through neurotrophic factors, transplantation of exogenous cells, cell replacement utilizing endogenous neural precursors, and direct reprogramming of resident glial cells into neurons^3,4^.

Substantial progress has been made in direct reprogramming of converting glial cells into neurons in vivo^5,6^. The generation of neuroblasts induced by retroviral expression of a dominant negative form of Olig2 in the injured cortex was first divulged by Buffo and his colleague^7^. From 2013 onwards, numerous studies have discovered that the expression of a single transcription factor (TF), such as NeuroD1 and Ascl1, is able to directly convert endogenous glial cells into neuroblasts or neurons^8–14^. Other researchers also stated the ability to the neuronal reconstruction of in-vivo-reprogrammed neurons, in particular damaged neural circuit of vision in blind mice and even in a Parkinson’s disease mouse model whose motor behavior was moderately corrected^15–17^. Despite the fact that all the studies mentioned above highlight the great value of in vivo reprogramming for disease modeling and cell therapy, the applications of reprogrammed neurons in other neuronal diseases or the basic integration of reprogrammed neurons into existing neural circuits are seldom characterized.

Proneural genes Ascl1 and Neurog2 play central roles in the acquisition of a neuronal identity during nervous system development and are widely used for direct neuronal reprogramming^18,19^. Our previous research revealed that Ascl1 alone efficiently converts astrocytes into functional neurons in vivo^13^. Neurog2, which is required for the expression of glutamatergic neurotransmitter phenotype in the embryonic neocortex, was reported to convert astrocytes into glutamatergic neurons efficiently in vitro^20,21^. Recent studies showed that Neurog2 is essential for in vivo reprogramming^22,23^. Along with Nurr1, Neurog2 can reprogram astrocytes to lamina-specific neurons with high efficiency^24^. NeuroD1, a downstream target of Neurog2, was suggested to convert reactive glial cells into functional neurons straightaway in vivo^11^.

In this study, we found that Neurog2 alone could efficiently convert astrocytes from central nervous system regions into neuronal cells in vivo, including midbrain, as well as intact and lesioned spinal cord. The majority of Neurog2-iNs in the midbrain are glutamatergic neurons, which can form synapses with local neurons. In the spinal cord, Neurog2-iNs can respond to diverse afferent inputs from dorsal root ganglion (DRG).

## Results

### Neurog2 converts dorsal midbrain astrocytes into functional neurons in vivo

**Fig. 1:**
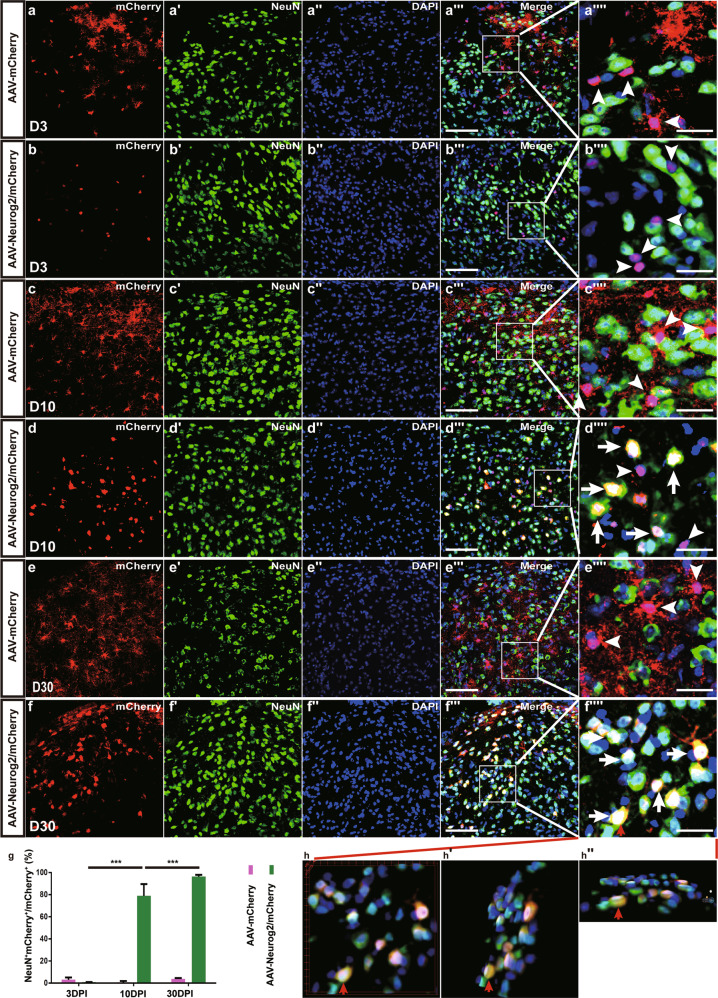
Neurog2 converts dorsal midbrain astrocytes into neurons in vivo. **a–b″″** Double staining of mCherry and NeuN on sections of the dorsal midbrain from WT mice that were infected with the control virus AAV–mCherry (**a**–**a″″**) or AAV-Neurog2/mCherry (**b**–**b″″**) on day 3. **c**–**d″″** Double staining of mCherry and NeuN on sections of the dorsal midbrain from WT mice that were infected with the control virus AAV–mCherry (**c**–**c″″**) or AAV-Neurog2/mCherry (**d**–**d″″**) on day 10. **e**–**f″″** Double staining of mCherry and NeuN on sections of the dorsal midbrain from WT mice that were infected with the control virus AAV–mCherry (**e**–**e″″**) or AAV-Neurog2/mCherry (**f**–**f″″**) on day 30. Panels (**a″″**, **b″″**, **c″″, d″″, e″″, f″″**) are higher magnifications of the boxed areas in (**a″′**, **b″′**, **c″′, d″′, e″′, f″′**), respectively. mCherry was not colocalized with NeuN (arrowheads). mCherry colocalized with NeuN (arrows). **g** The statistical data of astrocyte-to-neuron conversion efficiency induced by Neurog2 at different time points. A one-way ANOVA revealed a significant effect of group (F[5,17] = 493.2, *p* < 0.001), followed by Tukey’s multiple comparison test. *** Represents *p* < 0.001; scale bars: 50 μm (**a″′, b″′, c″′, d″′, e″′, f″′**) and 25 μm (**a″″**, **b″″**, **c″″, d″″, e″″, f″″**). **h**–**h″** 3D images of (**f″″**): (**h**) front view of (**f″″**), (**h′**) right-side view of (**h**) rotated 30°, (**h″**) bottom view of (**h**) rotated 30°. Red arrows in (**f″″, h, h′, h″**) show a typical view of the same cell.

Neurog2 is required for the differentiation of neural progenitors during development^21,25,26^, which is considered to be a potential candidate for reprogramming cell fates in vivo. To test the capability of astrocyte-to-neuron conversion induced by Neurog2, AAV-mCherry and AAV-Neurog2/mCherry were injected separately into the adult dorsal midbrain of wild-type (WT) adult mice. Immunostaining results showed that mCherry and Neurog2 were co-expressed at 3 days post infection (3 DPI) (Supplementary Fig. S1a–d). After staining mCherry with astrocyte marker S100β or neuronal marker NeuN, the colocalization between mCherry and astrocyte marker S100β (AAV-mCherry: 94.8 ± 2.8%, *n* = 6 mice; AAV-Neurog2/mCherry: 92.6 ± 5.6%, *n* = 3 mice; Supplementary Fig. S1e–f′) was observed in our experiment, but the colocalization between mCherry and NeuN was barely noticed (AAV-mCherry: 3.0 ± 2.1%, *n* = 6 mice, Fig. 1a–a″″, g; AAV-Neurog2/mCherry: 0.9 ± 0.1%, *n* = 3 mice, Fig. 1b–b″″, g) in the midbrain tissue infected with both AAV-mCherry vector and AAV-Neurog2/mCherry vector. The result suggested that gene expression of Neurog2 specifically targeted astrocytes rather than neurons under GFAP promoter at 3 DPI. At later time points, the vast majority of mCherry remained colocalizing with S100β (Supplementary Fig. S1g, g′ and data not shown), instead of colabeling with NeuN in tissues infected with AAV-mCherry vector at 10 DPI (0.7 ± 1.2%, *n* = 3 mice, Fig. 1c–c″″, g) or at 30 DPI (3.8 ± 0.8%, *n* = 6 mice; Fig. 1e–e″″, g). By contrast, mCherry became progressively colocalized with NeuN in tissues infected with AAV-Neurog2/mCherry, with the percentage of colocalization increasing to 78.9 ± 10.6% (Fig. 1d–d″″, g) at 10 DPI and to 96.3 ± 1.7% (Fig. 1f–f″″, g) at 30 DPI.

**Fig. 2:**
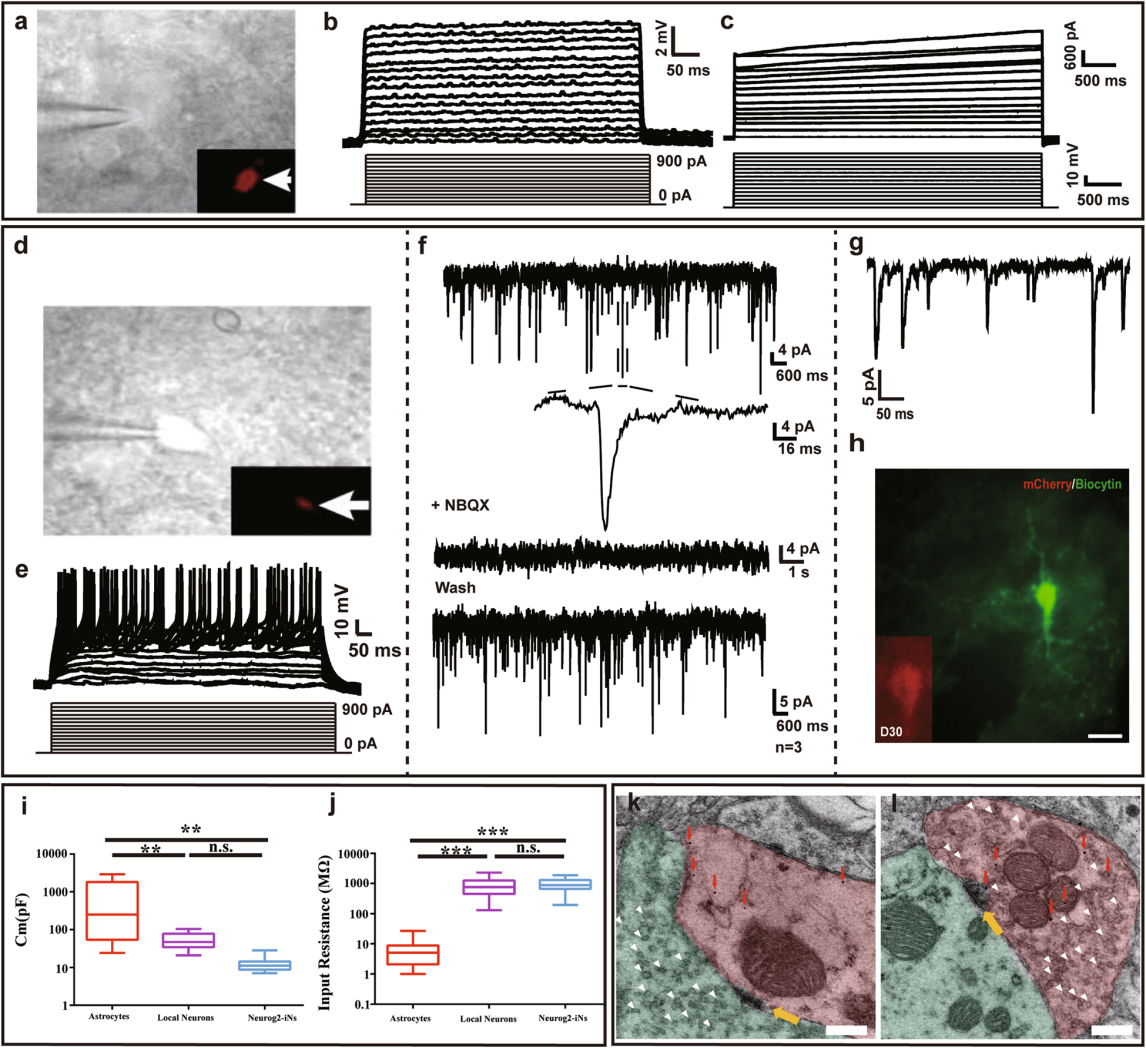
iN cells are functional neurons and can form synapse with local neurons in midbrain. **a–c** Membrane properties of an mCherry^+^ astrocyte recorded in an acute dorsal midbrain slice prepared from a WT mouse that was infected with the control virus AAV–mCherry at 30 DPI. Membrane voltages (**b**) and currents (**c**) were recorded in current- and voltage-clamp modes, respectively, in responses to the step current or voltage commands. Arrow indicates the cell recorded (**a**). **d–h** Membrane functions of iN cells (mCherry^+^, **d**) in slices of the dorsal midbrain that were prepared from WT mice infected with AAV–Neurog2/mCherry virus at 30 DPI. Membrane voltages (**e**) and membrane currents (**f**) were recorded in current- and voltage-clamp modes, respectively. The membrane currents were blocked with NBQX. **g** EPSCs were recorded in the mCherry+ cells of iN cells. **h** Double staining of Biocytin and mCherry on mCherry^+^ cells from AAV-Neurog2/mCherry-infected slices. Scale bar: 25 μm. **i** The comparison of membrane capacitance among AAV-mCherry-infected cells (red), local neurons (purple), and AAV-Neurog2/mCherry-infected cells (light blue). A one-way ANOVA revealed a significant effect of group (F[2,49] = 9.31, *p* < 0.001), followed by Tukey’s multiple comparison test. **j** The comparison of input resistance among AAV-mCherry-infected cells, local neurons and AAV-Neurog2/mCherry-infected cells. A one-way ANOVA revealed a significant effect of group (F[2,49] = 33.50, *p* < 0.001), followed by Tukey’s multiple comparison test. A total of 31 cells, 14 cells, and 15 cells were recorded in astrocytes, local neurons, and Neurog2-iNs, respectively. ** And *** represent 0.001 < *p* < 0.01 and *p* < 0.001, respectively; n.s. denotes not significant. **k, l** The fine structure of synapse between iN cells and local cells by immunogold electron microscopy. Brown-colored areas in (**k**) and (**l**) show iN cells (mCherry^+^, marked by gold particles, red arrows); light green areas (local neurons) represent presynaptic structure (**k**) and postsynaptic structures (**l**), respectively; White arrowheads indicate synaptic vesicles. Yellow arrows indicate connective buttons between induced neurons and host cells. Scale bar: 200 nm.

The functional properties of Neurog2-induced neuronal (iN) cells were examined by performing whole-cell recordings in acute brain slices in which the infected cells were identified by the expression of mCherry (Fig. 2a, d). Not any fire action potential (AP) was found in response to intracellular injection of step currents in the brain slices from mice infected with virus AAV–mCherry at 30 DPI (Fig. 2b, c). Moreover, cells from AAV–mCherry mice brain also exhibited a higher membrane capacity (871.47 ± 1039.15 pF, *n* = 31 cells; Fig. 2i) and a relatively low input resistance (7.67 ± 8.15 MΩ, *n* = 31 cells; Fig. 2j) at 30 DPI. These properties were quantitatively comparable with those of astrocytes^27^, which implies that the AAV–mCherry virus specifically targeted astrocytes in vivo, none of the physiological properties were changed under the control virus infection. On the contrary, most infected cells from AAV–Neurog2/mCherry-infected brain slices displayed both changes in the membrane potential and 93.75% multiple APs at 30 DPI (Fig. 2d, e and Supplementary Fig. S2e). In addition, inward (putative Na^+^) and outward (putative K^+^) currents of iN cells were caught in the voltage-clamp mode (Fig. 2d, e), excitatory postsynaptic currents (EPSCs) could be observed in all the cells recorded (Fig. 2g). We further demonstrated that iN cells can form synapses with local cells (Fig. 2f). Ancillary pharmacological experiments carried out at 30 DPI suggested that the excitatory glutamatergic synaptic inputs were received by iN cells whose synaptic transmission is mediated by AMPA receptor. Furthermore, the morphology of recorded iN cells showed a neuronal profile, as shown by the post-recording biocytin staining and the immunofluorescent staining (Fig. 2h). These results pointed out that Neurog2-induced neurons were physically integrated into the host circuits. Notably, the membrane capacitance (Fig. 2i) and the input resistance of iN cells were largely comparable with local neurons (Fig. 2j). Then the electrophysiological properties of Neurog2-iNs were analyzed after 10 days of virus injection, these induced cells were not only noted to exhibit the properties of immature neurons (Supplementary Fig. S2), but also develop the synapses with local cells (Fig. 2k, l; yellow arrows). We also found that Neurog2-induced neurons can act as both postsynaptic neurons (Fig. 2k) and presynaptic neurons, which was distinguished by synaptic vesicles (Fig. 2l; white arrowheads). The results indicated that Neurog2-induced neurons are physically integrated into host circuits.

At 30 DPI, iN cells no longer maintained astrocyte identity for which mCherry^+^s100β^+^ cells were barely visible in AAV-Neurog2/mCherry group (0.74 ± 1.24%, *n* = 3 mice; Supplementary Fig. S1h, h′). The density of s100β^+^ cells in the virus-infected area was similar in those uninfected areas nearby, which indicates that Neurog2 reprogramming did not result in the loss of local astrocytes (Supplementary Fig. S1i–m). Above results have shown that Neurog2 alone could efficiently convert dorsal midbrain astrocytes into functional neurons in vivo.

### The majority of Neurog2-iN cells in midbrain are glutamatergic neurons

**Fig. 3:**
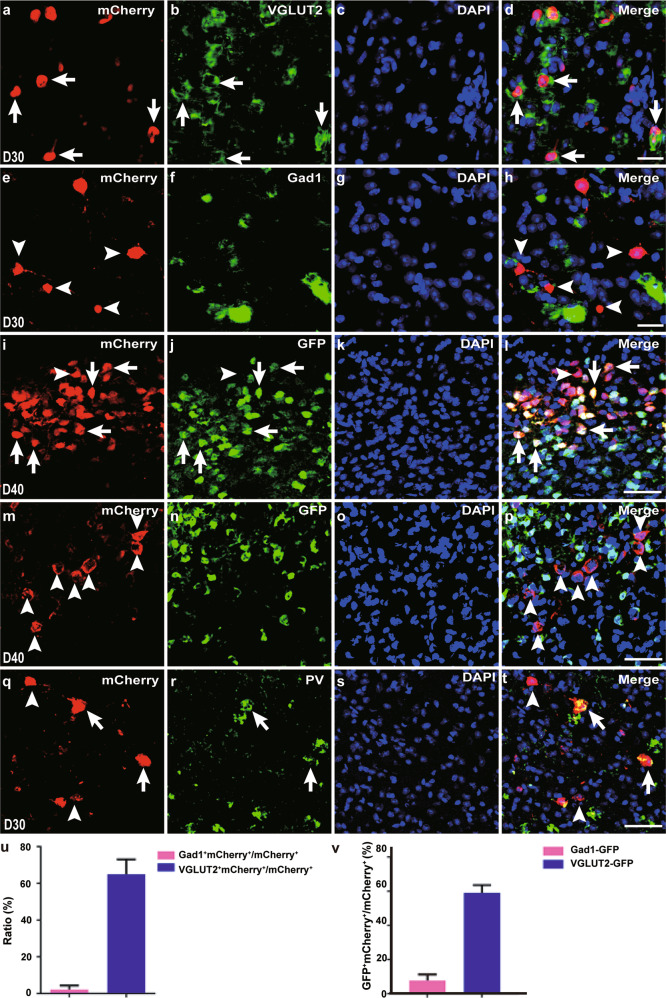
The majority of Neurog2-iN cells are glutamatergic neurons. **a–h** Double staining of mCherry protein (**a**, **e**) with VGLUT2 mRNA (**b**, green, pseudo-color) or Gad1 mRNA (**f**, green, pseudo-color) on sections of the dorsal midbrain from WT mice that were infected with virus AAV–Neurog2/mCherry at 30 DPI. Arrows in merged image (**d**) indicate mCherry^+^ cells can express VGLUT2. Arrowheads in merged image (**h**) indicate mCherry^+^ cells did not express Gad1. The nucleus was stained by DAPI (**c**, **g**). **i–p** Double staining of mCherry with GFP from VGLUT2-GFP mice (**i**–**l**) or GFP from Gad1-GFP mice (**m–p**) on sections of the dorsal midbrain that were infected with virus AAV-Neurog2/mCherry at 40 DPI. (**l**) and (**p**) are merged images from the mCherry signal (**i**, **m**), GFP signal (**j**, **n**), and DAPI (**k**, **o**), respectively. Arrows indicate mCherry^+^GFP^+^ cells and arrowheads indicate mCherry^+^GFP^-^ cells (**l**, **p**). **q–t** Represent mCherry (**q**) colabeled with PV (**r**) on sections of the dorsal midbrain from WT mice that were infected with virus AAV–Neurog2/mCherry at 30 DPI. Arrows in merged image (**t**) indicate mCherry^+^PV^+^ cells and arrowheads indicate mCherry^+^PV^-^ cells. The nucleus is stained by DAPI (**s**). **u**, **v** Statistical data indicate the percentages of colabeled cells from WT mice (**a–h**, **u)** and transgenic mice **(i–p**, **v**). (**a–h**) Confocal images 40x (oil); (**i–t**) Confocal images 20x. Scale bars: 25 μm (**a–h**) and 50 μm (**i–t**).

The neurotransmitter phenotype examination of Neurog2-iN cells in the midbrain was implemented next. Double staining of mCherry protein and VGLUT2 mRNA or Gad1 mRNA shown that Neurog2-iN cells expressed glutamatergic neuronal marker VGLUT2 (64. 97 ± 8.04%, *n* = 3 mice; Fig. 3a–d, u), but iN cells rarely expressed GABAergic neuronal marker Gad1 (2.26 ± 2.07%, *n* = 4 mice; Fig. 3e–h, u) at 30 DPI. We further adopted *VGLUT2-GFP* and *Gad1-GFP* mouse lines to examine the neurotransmitter phenotypes of iN cells, AAV-Neurog2/mCherry virus was injected into the dorsal midbrain of adult *VGLUT2-GFP* or *Gad1-GFP* mice. At 40 DPI, the colocalization of mCherry and GFP was observed in iN cells in the *VGLUT2-GFP* mice (59.03 ± 4.55%, *n* = 3 mice; Fig. 3i–l, v), whereas mCherry was found to be barely colocalized with GFP in *Gad1-GFP* mice (6.20 ± 3.56%, *n* = 8 mice; Fig. 3m–p, v). It has been reported that parvalbumin (PV) neurons in the dorsal midbrain are excitatory neurons and exhibit fast-spiking characteristics^28^. Double immunostaining showed that some of the Neurog2-iN cells were parvalbumin-positive cells (PV^+^, 17.85 ± 5.46%, *n* = 4 mice; Fig. 3q–t) with fast-spiking electrophysiological properties (Fig. 2e), which are similar to those characteristics of endogenous PV neurons in the dorsal midbrain as stated in previous research^28^. It is noteworthy that Neurog2 has been used for converting fibroblasts into dopaminergic and serotonergic neurons^29,30^. Besides, Neurog2-iN cells did not express molecular markers for dopaminergic and serotonergic neurons including tyrosine hydroxylase and serotonin, respectively, in our model (data not shown). Thus, our results prove that Neurog2 alone can specifically generate substantial glutamatergic neurons from midbrain astrocytes in vivo.

### Neurog2-iN cells do not pass through a proliferative stage

In order to further determine whether Neurog2 converts astrocytes into neurons in vivo, we used astrocyte-specific mouse line Aldh1l1-Cre. Cre-inducible AAV viruses, AAV-FLEX-NLSGFP, and AAV-FLEX-Neurog2/GFP that contains FLEX switch sequence response to Cre in the AAV vector, were injected into the dorsal midbrain of *Aldh1l1-Cre* mice. The colocalization of GFP with S100β (AAV-FLEX-NLSGFP: 93.94 ± 2.61%, *n* = 4 mice, Supplementary Fig. S3a, a′; AAV-FLEX-Neurog2/GFP: 94.56 ± 4.11%, *n* = 3 mice; Supplementary Fig. S3b, b′) was found at 3 DPI, but GFP barely colocalized with NeuN (AAV-FLEX-NLSGFP: 4.43 ± 2.64%, *n* = 3 mice; Supplementary Fig. S3c, c′; AAV-FLEX-Neurog2/GFP: 1.38 ± 2.03%, *n* = 3 mice Supplementary Fig. S3e, e′) in both AAV-FLEX-NLSGFP- and AAV-FLEX-Neurog2/GFP-infected mice. At 30 DPI, GFP^+^ cells hardly expressed NeuN in the dorsal midbrain tissues infected with AAV-FLEX-NLSGFP (AAV-FLEX-NLSGFP: 3.80 ± 2.70%, *n* = 3 mice; Supplementary Fig. S3d, d′). However, the majority of GFP^+^ cells colabeled with NeuN in tissues infected with AAV-FLEX-Neurog2/GFP (AAV-FLEX-NLSGFP: 79.43 ± 12.79%, *n* = 3 mice; Supplementary Fig. S3f, f′). Thus, the conditional expression of Neurog2 is also capable of converting astrocytes into neurons. To further shape the morphology and examine the expression of immediate early genes of Neurog2-iNs, we simultaneously injected AAV-CAG-FLEX-Ngn2-GFP and AAV-hSyn-DIO-ChrimsonR-tdTomato viruses into the midbrain of Aldh1l1-cre mice. After 30 days, virus-infected cells show classic neuronal morphology (Supplementary Fig. S3g). By laser stimuli, Neurog2-iNs can be activated and they express immediate early genes c-Fos (Supplementary Fig. S3h–h″″).

To test our hypothesis that Neurog2-induced neurons could be converted from astrocytes by a direct fate switch without a proliferation phase, mice injected with AAV-mCherry or AAV-Neurog2/mCherry in the dorsal midbrain were continually treated by intraperitoneal injection of 5-bromo-2′-deoxyuridine (BrdU) at 3–30 DPI^13^. We found that mCherry barely colocalized with BrdU in the midbrain tissue infected with control AAV-mCherry (1.00 ± 2.24%, *n* = 5 mice; Supplementary Fig. S3i) or AAV-Neurog2/mCherry (1.32 ± 1.45%, *n* = 6 mice; Supplementary Fig. S3j) at 30 DPI. These results collectively demonstrated that Neurog2 converts midbrain astrocytes into neurons without passing through a proliferation phase.

### Neurog2 directly converts astrocytes from intact spinal cord into functional neurons in vivo

**Fig. 4:**
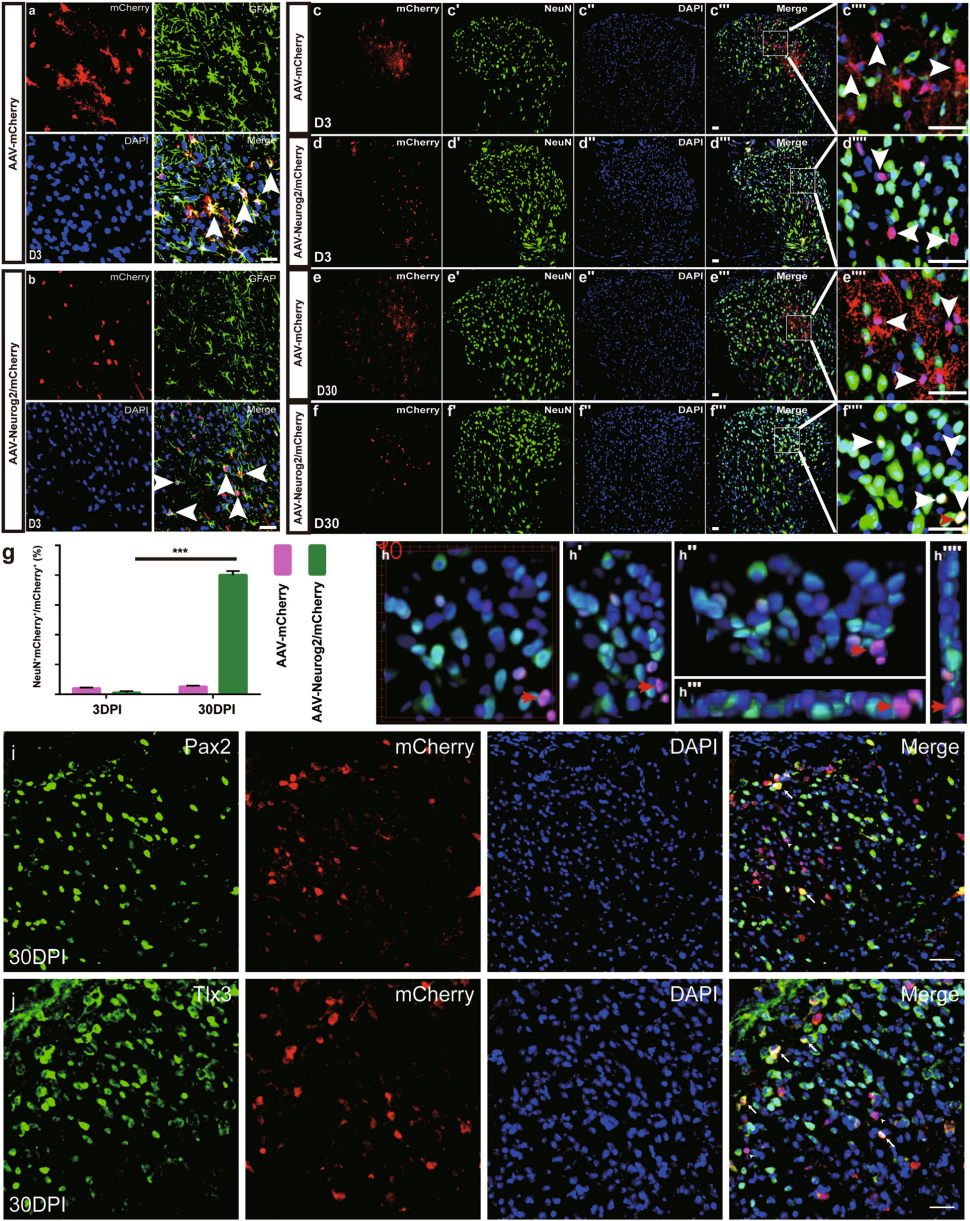
Neurog2 converts astrocytes into neurons from intact mouse dorsal spinal cord in vivo. **a**, **b** Double staining of mCherry and GFAP on transections of the dorsal spinal cord from WT mice infected with AAV-mCherry (**a**) or AAV-Neurog2/mCherry (**b**) at 3 DPI. Upper left, upper right, bottom left, and bottom right represent mCherry, GFAP, DAPI, and merged images, respectively. Arrows in merged images indicate mCherry^+^GFAP^+^ cells. Scale bar: 20 μm (**a**, **b**). **c–d″″** Double staining of mCherry and NeuN on transections of the dorsal spinal cord from WT mice that were infected with the control virus AAV–mCherry (**c**–**c″″**) or AAV-Neurog2/mCherry (**d–d″″**) on day 3. **e–f″″** Double staining of mCherry and NeuN on sections of the dorsal spinal cord from WT mice that were infected with the control virus AAV–mCherry (**e–e″″**) or AAV-Neurog2/mCherry (**f-f″″**) on day 30. Panels (**c″″, d″″, e″″, f″″**) are higher magnifications of the boxed areas in (**c″′, d″′, e″′, f″′**), respectively. mCherry was not colocalized with NeuN (arrowheads), mCherry colocalized with NeuN (arrows). Arrows and arrowheads in (**c″″, d″″, e″″, f″″**) represent mCherry^+^NeuN^+^ cells and mCherry^+^NeuN^-^ cells, respectively. Scale bars: 50 μm (**c″′, d″′, e″′, f″′**) and 25 μm (**c″″, d″″, e″″, f″″**). **g** The statistical data of astrocyte-to-neuron conversion efficiency induced by Neurog2 at different time points. A one-way ANOVA revealed a significant effect of group, followed by Tukey’s multiple comparison test. ***Represents *p* < 0.001; **h–h″″** 3D images of (**f″″″)**: (**h**) front view of (**f″″**), (**h′**) left side of view of (**h**) rotated 30°, (**h″**) top view of (**h**) rotated 30°, (**h″′**) bottom view of (**h**), (**h″″**) right view of (**h**). The red arrows in (**f″″, h, h′, h″, h″′, h″″**) show a typical view of the same cell. **i, j** Immunostaining of Pax2 (**i**) and Tlx3 (**j**) on sections of spinal cord at 30 DPI.

We further study whether Neurog2 could converts astrocytes into neurons in intact and lesioned spinal cord in vivo. Firstly, AAV-mCherry and AAV-Neurog2/mCherry viruses were delivered individually into the dorsal horn at thoracic 8–10 (T8–T10) of adult mice. At 3 DPI, both vectors targeted astrocytes, which were indicated by mCherry colabeling with astrocyte marker GFAP (AAV-mCherry: 92. 29 ± 5.26%, *n* = 4 mice; AAV-Neurog2/mCherry: 90.31 ± 3.19%, *n* = 4 mice; Fig. 4a, b), rather than neuronal marker NeuN (AAV-mCherry: 4.04 ± 0.71%, *n* = 3 mice, Fig. 4c–c″″, g; AAV-Neurog2/mCherry: 1.08 ± 1.86%, *n* = 3 mice; Fig. 4d–d″″, g). At 30 DPI, mCherry^+^ cells did not express NeuN in control AAV-mCherry-infected tissues (4.04 ± 0.71%, *n* = 3 mice; Fig. 4e–e″″, g). On the contrary, mCherry colocalized with NeuN in tissue infected with AAV-Neurog2/mCherry, with a percentage up to 80.11 ± 5.42% (*n* = 4 mice; Fig. 4f–f″″, g, h–h″″). We also found that Neurog2-induced neuronal cells consist of both glutamatergic (50.9 ± 8.8%, *n* = 3 mice; Fig. 4j) and GABAergic (38.5 ± 8.3%, *n* = 3 mice; Fig. 4i) neurons.

**Fig. 5:**
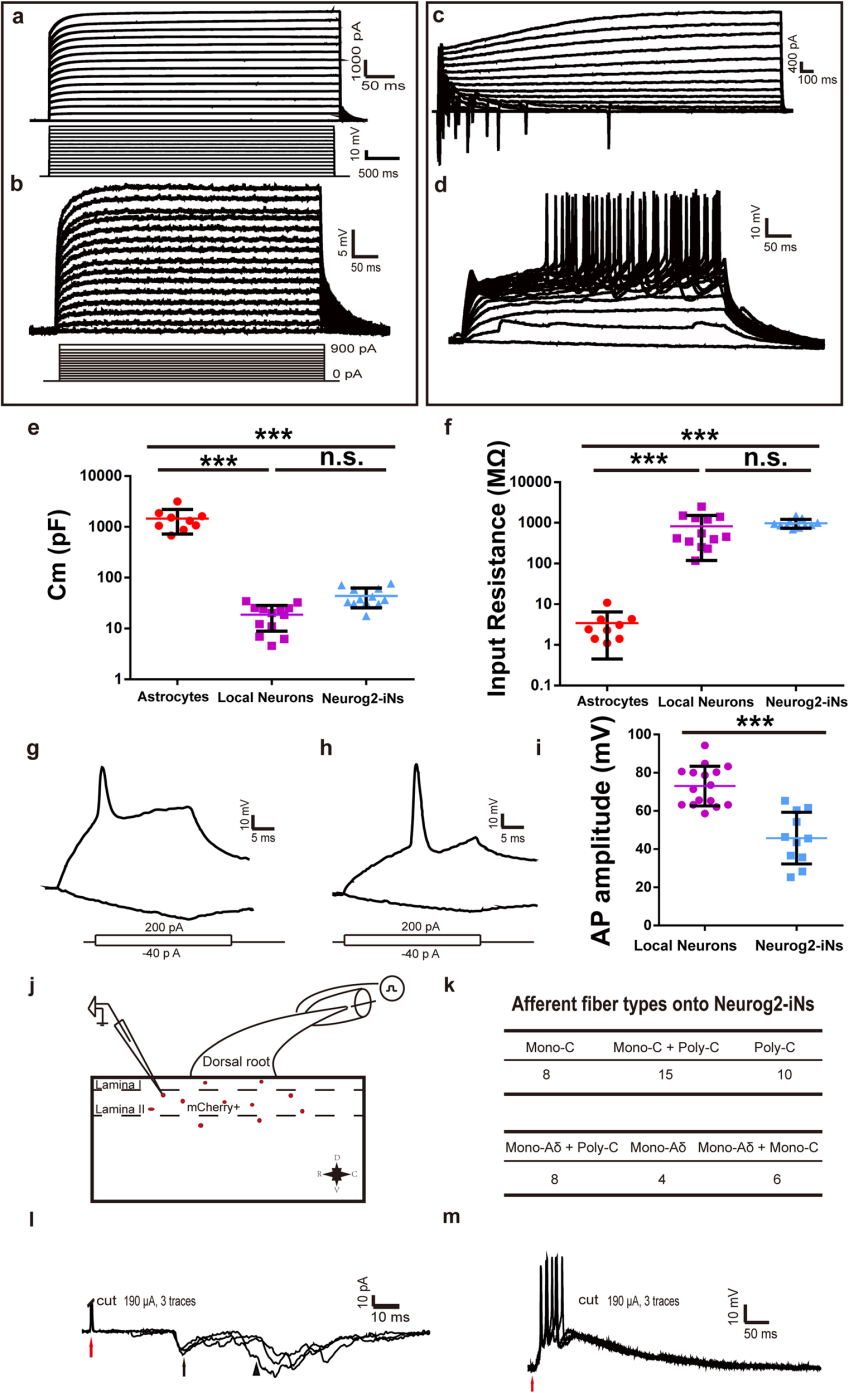
Neurog2-iN cells exhibit mature electrophysiological properties and receive peripheral inputs from dorsal root ganglion. **a**, **b** Membrane properties of an mCherry^+^ astrocyte recorded in an acute dorsal spinal cord slice prepared from a WT mouse infected with the control virus AAV–mCherry at 30 DPI. Membrane currents (**a**) and voltages (**b**) were recorded in voltage- and current-clamp modes, respectively, in responses to the step current or voltage commands. **c**, **d** Membrane functions of iN cells (mCherry^+^) in slices of the dorsal spinal cord that were prepared from WT mice infected with AAV–Neurog2/mCherry at 30 DPI. Membrane currents (**c**) and membrane voltages (**d**) were recorded in voltage- and current-clamp modes, respectively. **e** The comparison of membrane capacitance among AAV-mCherry-infected cells (red), local neurons (purple), and AAV-Neurog2/mCherry-infected cells (light blue). A one-way ANOVA revealed a significant effect of group (F[2,31] = 48.02, *p* < 0.001), followed by Tukey’s multiple comparison test. **f** The comparison of input resistance among AAV-mCherry-infected cells, local neurons and AAV-Neurog2/mCherry-infected cells. A one-way ANOVA revealed a significant effect of group (F[2,31] = 11.54, *p* < 0.001), followed by Tukey’s multiple comparison test. **g, h** Two kinds of representative traces of APs induced by depolarization currents of Neurog2-iN cells (mCherry^+^) in the slice of spinal cord. **i** The comparison of AP amplitude (mV) of recorded cells between local neurons and Neurog2-iN cells. Statistical analysis was performed using the two-tailed *t*-test method. **j** Schematic showing relative positions of recorded mCherry^+^ cells. D, V, R, and C in (**j**) represent dorsal, ventral, rostral, and caudal, respectively. **k** The table is a summary of inputs from DRG in 51 recorded mCherry^+^ cells from 5 mice. **l** Typical traces of C-fiber-evoked mono-C and poly-C EPSCs onto mCherry^+^ cells. **m** Typical traces of Aδ-fiber-evoked EPSPs and APs showing Aδ fiber inputs onto mCherry^+^ cells. *** Represents *p* < 0.001, n.s. denotes not significant.

Secondly, the functional properties of iN cells were tested by performing whole-cell recordings in acute spinal cord slices obtained from virus-infected mice at 30 DPI. The infected cells were identified by the expression of mCherry. In slices from mice infected with the control virus AAV–mCherry, we found that infected cells exhibit no inward current in the voltage-clamp mode (Fig. 5a) and no AP (Fig. 5b) in response to intracellular injection of step currents. Those cells also exhibited a higher membrane capacity (Fig. 5e) along with a relatively low input resistance (Fig. 5f). These properties were also quantitatively comparable with those of astrocytes^27^. In slices from mice infected with AAV–Neurog2/mCherry, most infected cells displayed inward (putative Na^+^) and outward (putative K^+^) currents in the voltage-clamp mode (Fig. 5c) and most recorded cells (9/11) could fire multiple APs (Fig. 5d). However, the iN cells induced by Neurog2 had a relatively lower AP amplitude compared with that of local neurons (Fig. 5g–i), which was different from the observation in midbrain iN cells (all recorded cells had normal AP amplitude). The membrane capacitance and the input resistance of iN cells were largely comparable with that of local neurons (Fig. 5e, f). These results indicate that Neurog2 could convert dorsal spinal cord astrocytes into functional neurons in adult mice.

Finally, to determine whether Neurog2-iN cells can be functionally integrated into host circuits, we performed an electrophysiological experiment on the spinal cord. Mice were injected with AAV-Neurog2/mCherry virus at lumbar L1-L2 (longer free fiber facilitates stimulation of DRG) and were sacrificed for recording at 30 DPI. We found that the majority of mCherry^+^ cells located at the superficial layer (Fig. 5j), 51 cells were randomly picked from 5 mice to perform the recording in this experiment. After giving stimuli at DRG, we noticed that 8 cells can receive mono-C fiber inputs, 15 cells can receive mono-C + poly-C fiber inputs, 8 cells can receive mono-Aδ + poly-C fiber inputs, and the rest 10, 4, and 6 cells can receive poly-C, mono-Aδ, and mono-Aδ + mono-C inputs, respectively (Fig. 5k). Red arrows denote the stimulation artifacts, black arrow and arrowhead explicate mono-C- and poly-C-evoked EPSCs to one cell, respectively (Fig. 5l). Interestingly, 15 of the recorded cells can fire APs by stimulating the dorsal roots, which suggested that mCherry^+^ cells can potentially generate outputs to local circuits (Fig. 5m). Those results indicated that Neurog2-iN cells can be activated by peripheral afferents and well integrated into local circuits.

### Neurog2 directly converts astrocytes from lesioned spinal cord into neuronal cells in vivo

**Fig. 6:**
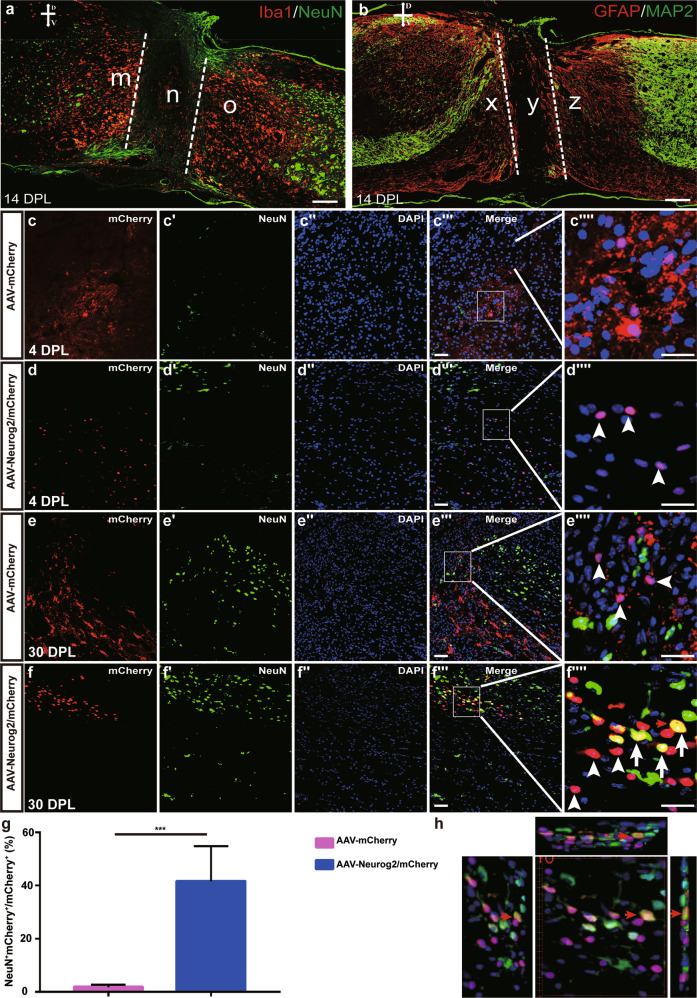
Neurog2 converts astrocytes of lesioned spinal cord into neuronal cells in vivo. **a** Double staining of NeuN and Iba1 on horizontal sections of the lesioned spinal cord at 14 DPL. Iba1 acts as microglia marker and represents the intensity of the immune response. **b** Double staining of GFAP and MAP2 on horizontal sections of the lesioned spinal cord at 14 DPL. MAP2 and GFAP indicate neuronal fibers and astrocytes, respectively. m, o, x, z regions in (**a**, **b**) denote neuron-lost areas adjacent to lesion sites (n, y). **c–d″″** Horizontal images of the lesioned spinal cord shown double staining of mCherry and NeuN on slices from mice infected with the control virus AAV–mCherry (**c**–**c″″**) or AAV-Neurog2/mCherry (**d–d″″**) on day 4. **e–f″″** Horizontal images of the lesioned spinal cord show double staining of mCherry and NeuN on slices from mice infected with the control virus AAV–mCherry (**e–e″″**) or AAV-Neurog2/mCherry (**f–f″″**) on day 30. Panels (**c″″, d″″, e″″, f″″**) are higher magnifications of the boxed areas in (**c″′, d″′, e″′, f″′**), respectively. mCherry was not colocalized with NeuN (arrowheads), mCherry colocalized with NeuN (arrows). Arrows and arrowheads in (**c″″, d″″, e″″, f″″**) represent mCherry^+^NeuN^+^ cells and mCherry^+^NeuN^-^ cells, respectively. Scale bars: 50 μm (**c″′, d″′, e″′, f″′**) and 25 μm (**c″″, d″″, e″″, f″″**). **g** The statistical data of astrocyte-to-neuron conversion efficiency induced by Neurog2 at 30 DPI. A two-tailed *t*-test was applied. *** Represents *p* < 0.001; **h** 3D images of (**f″″″)** view: left view with 30° rotation (left), front view (middle), top-side view with 30° rotation (top), and right-side view (right). The red arrows in (**f″″, h**) show a typical view of the same cell.

Spinal cord injury (SCI) leads to irreversible neuronal loss and glial scar formation, in vivo reprogramming of endogenous astrocytes to neurons might be a potential strategy for cellular regeneration after SCI^31^. A spinal cord lesion model was generated by a complete transection of the spinal cord at the T8–T10 level. At 14 days post lesion (DPL), neurons and neuronal fibers were lost in the injury core region, whereas microglia and astrocytes were enriched adjacent to the cut sites (Fig. 6a, b). To investigate whether Neurog2 could convert spinal cord astrocytes into neurons in SCI mice, AAV-mCherry and AAV-Neurog2/mCherry virus was injected individually into the parenchyma of the severely injured spinal cord immediately after lesion. The four injection sites were 1.0 mm away from the lesion core on each side. Histological analyses have shown that AAV-mCherry vector displayed an astrocyte expression pattern, mCherry colabeled with astrocyte marker GFAP (data not shown) but not neuronal marker NeuN at 4 DPL and 30 DPL (1.87 ± 2.06%, *n* = 3 mice; Fig. 6c–c″″, g). AAV-Neurog2-mCherry virus targeted expression of mCherry in GFAP^+^ glial astrocytes (data not shown) rather than NeuN^+^ neuronal cells at 4 DPL, the infected astrocytes were converted into NeuN^+^ cells in the lesioned spinal cord at 30 DPL (Fig. 6d–d″″, f–f″″, h). The conversion efficiency of astrocyte-to-neuron was 41.62 ± 22.82% at 30 DPL (Fig. 6g). Take it as a whole, these results indicated that Neurog2 could convert astrocytes in the injured spinal cord into neurons as well.

## Methods

### Mouse strains

The generation of glutamic acid decarboxylase 67*(Gad67)-GFP* knock-in mice was described previously^32^. The mouse strains *VGLUT2-GFP* [stock Tg(Slc17a6-EGFP)] and Aldh1l1-Cre were obtained from the Mutant Mouse Regional Resource Center (MMRRC)^33,34^. *Gad67–GFP* and *VGLUT2-GFP* mice were identified with the primers 5′-GCACGACTTCTTCAAGTCCGCCATGCC-3′ and 5′-GCGGATCTTGAAGTTCAC CTTGATGCC-3′, which were used for detecting the GFP element. The Aldh1l1-Cre was identified with primers 5′-GCCTGCATTACCGGTCGATGC-3′ and 5′-CAGGGTGTTATAAGCAATCCCC-3′, which were used for detecting the Cre cassette. WT mice were C57BL and were obtained from SLAC Laboratory. All animal procedures are contained in protocols reviewed and approved by the institutional ethics and safety guidelines (Institutional Animal Welfare and Ethics Committee, First Affiliated Hospital of Bengbu medical college, Anhui, China) and the Animal Care Committee at the Institute of Neuroscience, Shanghai Institutes for Biological Sciences, Chinese Academy of Sciences (Reference NA-100426).

### Plasmid construction and adeno-associated virus (AAV) packaging

We generated GFAP-Neurog2-adeno-associated virus (AAV-Neurog2/mCherry, 2.2 Kb hGFAP Promoter) vectors by replacing Ascl1 of AAV-Ascl1/mCherry vector with mouse Neurog2 full-length cDNA (NCBI: NM_009718)^13^. Neurog2 was cloned into the vector AAV–FLEX–Arch–GFP (Addgene: #22222) to generate AAV–FLEX–Neurog2/GFP. The viral vectors AAV-mCherry and AAV-FLEX-NLSGEP were described previously^13^. Recombinant AAV stocks, serotype 8, were produced in our lab according to standard procedure^35^. Plasmids were co-transfected into HEK293T cells and supernatant was collected for virus isolation at 72 h post infection. Optiprep density gradients were employed to purify the viruses and Amicon Ultra-15 centrifugal filter unit was further applied for concentration. Purified AAV viruses were titered using a quantitative PCR-based method and were qualified by transmission electron microscope. AAV-hSyn-DIO-ChrimsonR-tdTomato virus was directly purchased from Shanghai Taitool Bioscience Co. Ltd.

### Immunostaining

Immunostaining on cryostat sections were performed as described previously^36^. Double staining that combined in situ hybridization with immunostaining on cryostat sections was performed as described previously^37^. For double staining that combined the in situ hybridization with immunostaining, the former was performed firstly with a lower proteinase K concentration (2 μg/ml) and shorter digesting time (5 min). After color development with nitro blue tetrazolium/5-bromo-4-chloro-3-indolyl-phosphate as substrates, immunostaining procedures to detect mCherry protein (1:200) were performed. VGLUT2 and Gad1 probes have been described previously and VGLUT1 probe are designed to target the full length of the cDNA^38^. To generate the confocal images, the bright-field images of in situ hybridization signals were converted into pseudo-green fluorescent color and then merged with the fluorescent images in ImageJ (Developed by NIH). Primary antibodies were as follows: mouse anti Neurog2 (1:500; MAB3314; R&D), mouse anti-GFAP (1:1000; MAB360; Millipore), chicken anti-GFP (1:1000; A10262; Invitrogen), mouse anti-NeuN (1:100; MAB377; Millipore), rabbit anti-NeuN (1:500, ABN78, Millipore), rat anti-RFP (1:200; 5f8-100; ChromoTek), rabbit anti-Discosoma red (1:500; 632496; Clontech), mouse anti-S100β (1:1000; S2532; Sigma), rabbit anti-doublecortin (DCX; 1:500; ab77450; Abcam), and mouse anti-BrdU (1:200; B2531; Sigma). FITC-, Cy3-, and Cy5-conjugated secondary antibodies were obtained from Jackson ImmunoResearch.

### Stereotactic injection of AAV virus

After the anesthetization with ketamine (100 mg/kg, i.p.)/xylazine (10 mg/kg, i.p.), viruses were injected into the dorsal midbrain according to the mouse brain atlas at the following coordinates:^39^ for the midbrain, anteroposterior, −3.3 and −4.1 mm; mediolateral, 0.5 mm; dorsoventral, −1.0 mm angled 90° toward the midline in the coronal plane. For the spinal cord, after performing laminectomy to expose the dorsal surface of the T8–T10 segment (for Fig. 5j–m, viruses were injected at L1–L2), viruses were injected into the dorsal surface with 30° angle and 0.2–0.3 mm depth. Virus stocks were diluted to 3–5 × 10^12^ vg/ml, and 0.5 μl virus would be injected with a speed of 100 nl/min. After feeding for days, the tissues were collected for immunostaining or slice recording.

Mice were pre-injected intraperitoneally with a mixture of midazolam (5 mg/kg), fentanyl (0.05 mg/kg), and medetomidine (0.5 mg/kg) in the optogenetic assay, their heads were fixed in stereotaxic apparatus after they were fully unresponsive to toe-pinch. A craniotomy (~1 mm diameter) was made above the dorsal midbrain (anteroposterior −3.3 mm, mediolateral 0.5 mm), virus was microinjected into the dorsal midbrain using a custom microinjector and microinfusion pump (PHD 2000, Harvard Apparatus). Fibers were secured in place with dental acrylic adhered to skull screws. Mice were connected to a 635-nm DPSS laser (Shanghai Laser) via fiber-optic cables and placed inside the testing chamber for optogenetic stimulation. Optical stimulation was triggered by Arduino. Stimulation parameters were consistent within a session, but the order of stimulation was semi-randomized between mice.

### Brain slice preparation

Brain slices were prepared typically ~1 month after viral injection as described previously with some modifications^13^. Mice were anesthetized with sodium pentobarbital (50 mg/kg, i.p.; sigma).The cardiac perfusion was performed with ice-cold artificial cerebrospinal fluid (aCSF) (in mM: 125 NaCl, 3 KCl, 2 CaCl_2_, 2 MgSO_4_, 1.25 NaH_2_PO_4_, 1.3 Na^+^-ascorbate, 0.6 Na^+^-pyruvate, 26 NaHCO_3_, and 11 glucose, at pH = 7.4) saturated with 95% O_2_ and 5% CO_2_. The brain was rapidly dissected and cut into 300 μm slices and incubated in a chamber containing aCSF at room temperature for about 1 h before recording.

#### Spinal cord slice preparation

Mice were anesthetized with ketamine (100 mg/kg, i.p.)/xylazine (10 mg/kg, i.p.), and the cardiac perfusion were performed with ice-cold cutting solution (in mM: 102 choline chloride, 2.5 KCl, 1.2 NaH_2_PO_4_, 30 NaHCO_3_, HEPES 20, 25 Glucose, 5 Na^+^-ascorbate, 2 Thiourea, 3 Na^+^-pyruvate, 10 MgSO_4_, 0.5 CaCl_2_, adjusted pH to 7.35−7.45 with Tris-Base, 300–310 mOsm), saturated with 95% O_2_ and 5% CO_2_. The vertebral plates were isolated, and the laminectomy were performed quickly. The L1–L5 Segments were dissected and embedded within the agarose and glued to the vibratome (VT-1200S, Leica), 400–500 μm sagittal slices attached with dorsal roots were cut and transferred to aCSF (in mM: 125 NaCl, 3 KCl, 2 CaCl_2_, 2 MgSO_4_, 1.25 NaH_2_PO_4_, 1.3 Na^+^-ascorbate, 0.6 Na^+^-pyruvate, 26 NaHCO_3_, and 11 glucose, pH 7.35−7.45, 300–310 mOsm) saturated with 95% O_2_ and 5% CO_2_ to recover at room temperature (25 °C) for at least 1 h before recording.

#### Brain slice recording

For mCherry^+^ cells, whole-cell recordings were performed using Multiclamp 700B patch-clamp amplifier (Molecular Devices) as described before^13^, and the chamber was constantly perfused with a bath solution containing the following (in mM): 125 NaCl, 3 KCl, 2 CaCl_2_, 2 MgSO_4_, 1.25 NaH_2_PO_4_, 1.3 Na^+^-ascorbate, 0.6 Na^+^-pyruvate, 26 NaHCO_3_, and 11 glucose, at pH 7.4, 290−310 mOsm, saturated with 95% O_2_ and 5% CO_2_. Patch pipettes were pulled from borosilicate glass (3–5 MΩ) and filled with a pipette solution consisting of (in mM): 130 K-gluconate, 20 KCl, 10 HEPES, 0.2 EGTA, 4 Mg_2_ATP, 0.3 Na_2_GTP, and 10 Na_2_-phosphocreatine, at pH 7.3 (290−310 mOsm). For the morphological reconstruction experiment, biocytin was included in the internal solution. To evoke currents, step voltages (500 ms, 10 mV step) from −110 to 60 mV were applied in the voltage-clamp mode. To evoke membrane potential deflections, step currents (500 ms duration) were injected in the current-clamp mode. Data were collected using pClamp 10 software (molecular Devices) digitized at 20–100 kHz, and analyzed with Clampfit 10.

#### Spinal cord slice recording

Whole-cell patch-clamp recordings were made from mCherry^+^ cells using glass pipettes with a resistance of 2–5 MΩ from borosilicate glass (Sutter, USA), which were made by P-97 horizontal micropipette puller (Sutter). Internal solution (in mM) was as follows: 130 K-gluconate, 20 KCl, 10 HEPES, 0.2 EGTA, 4 Mg_2_ATP, 0.3 Na_2_GTP, and 10 Na_2_-phosphocreatine, at pH = 7.3, Osmotic pressure at 290–310 mOsm.

Primary afferent inputs were evoked by stimulating L4 or L5 dorsal root through suction electrode (Sutter) with an isolator (AMPI). The root length was 8–10 mm. The intensity of stimulation was controlled to selectively stimulate Aβ/Aδ and C fibers, and conduction velocity (CV) was used to distinguish corresponding evoked EPSCs. CVs were determined by calculating the ratio between the latency and the length between the suction electrode and the dorsal entry zone. A stimulation intensity of ≤25 μA with a CV ≥ 2 m/s was considered to correspond to Aβ-fiber activity, and a stimulation intensity of 50–100 μA with a CV at 0.8–1 m/s was considered to correspond to Aδ-fiber activity, whereas a stimulation intensity of ≥100 μA with a latency ≤0.6 m/s was considered to correspond to C-fiber activity. Mono-Aδ or C-eEPSCs were judged by their response to high-frequency stimulation at 2 or 1 Hz, as reported previously^40^. By holding the membrane potential at −70 mV, eEPSCs can be detected and current-clamp mode at the resting membrane potential was used to record eEPSPS and dorsal root stimulation-evoked AP output. Data were acquired with pClamp9.2 (Molecular Devices) using an AxonMultiClamp 700A amplifier (Molecular Devices), filtered at 5 kHz (low pass), and digitized at 20–100 kHz (Digidata 1322 A; Molecular Devices). Before recording, the junction potential was corrected. The data analysis was performed with the Clamfit 10. All chemicals were purchased from Sigma, and all recordings were performed at room temperature.

### Immunogold electron microscopy

Mice were transcardially per­fused with 4% PFA and 0.2% glutaraldehyde in 0.1 M PBS. Brains were removed after perfusion and were immersed in the same fixation solution for 12 h at 4 °C. Coronal sections (150 μm thick) were sectioned using a vibratome (Leica VT1200 S). The brain sections containing dorsal midbrain areas were post-fixed in 0.5% osmium tetroxide and then dehydrated in ethanol series, and finally embedded in EMBed-812 (14120, EMS, USA) for 2 days. The ultrathin sections (90 nm) were cut and collected on nickel grids (200 mesh). For immune-labeling, the ultrathin sections were incubated with primary antibodies (rabbit anti-DsRed, 1:200) for 48 h at 4 °C, followed by incubation with goat anti-Rabbit IgG with gold conjugates (2004, Nanoprobes, 1:50) for 2 h at room temperature. The sections were then incubated with 1% glutaraldehyde to fix the gold particles. Slices were incubated with methanolic uranyl acetate and lead citrate before observing with a JEOL JEM-1230 electron microscopy.

### Construction of mice whole-transection spinal cord injury model

Surgeries were conducted under deep anesthesia using a combination of ketamine (100 mg/kg, i.p.) and xylazine (10 mg/kg, i.p.). Briefly, a laminectomy was performed to expose the entire dorsal surface of the T8–T10 segment^41^. A whole transection was then introduced by cutting using fine Venus Scissors and calibrated forceps, with visual verification to ensure complete transection ventrally and laterally. After the surgery, animals were returned to their home cages and received manual bladder expression twice on a daily basis until reflexive bladder control returned.

### Data analysis

Data were presented as mean ± SEM, two-tailed *t*-test was used to determine statistical significance between the two groups. One-way ANOVA followed by Tukey’s multiple comparison test was applied for the comparison among groups in the same graph. All conclusions are based on double blind experiments. The *p*-value less than 0.05 was considered statistically significant.

## Discussion

In this study, the single TF Neurog2 is validated to accomplish endogenous astrocyte-to-neuron conversion in the adult midbrain and the spinal cord. Neurog2-reprogrammed midbrain neurons could express neuronal marker, exhibit excitatory neuron identity, fire APs, receive synaptic inputs, and form synapses with preexisting neurons. Neurog2-iN cells in the spinal cord, which share the same electrophysiological features as mature neurons, are identified to be further functionally integrated into local circuits that respond to DRG stimuli.

The expression level of Neurog2 is a critical point for successful neuronal reprogramming. Recent studies convey that Neurog2 alone is capable of reprogramming astrocytes into NeuN+ cells in vivo^22,23^ with around 2% neuronal induction efficiency. We performed this study by ectopic expression of Neurog2 in astrocytes with AAV delivery system and successfully achieved astrocyte-to-neuron conversion in vivo with much higher efficiency (Fig. 1e, f, g′). Previous work had shown that a number of factors would determine the outcome of neuronal reprogramming, particularly with regard to cell death, oxidative stress, delivery system, and the host environment^22,23,42^. Retrovirus was once described to be a tool of Neurog2 overexpression in dividing cells in 2013 and 2016^22,23^. In this study, AAV vector was used to express Neurog2 under the direct control of a human GFAP promoter for the specific astrocytes infection. Different infected cells may contribute to different levels of efficiency of neuronal induction. Grande^23^ claimed that only approximately 17% of infected cells retained Neurog2 expression at 28DPI or later, which suggested that transgene was silenced in their virus delivery system. Excitingly, 90% of infected cells were determined to continuously express Neurog2 at 30DPI (Supplementary Fig. S1k), which intimates the long-term expression of Neurog2 in our study.

The cellular source of Neurog2-reprogrammed cells is another critical point for this research. Three crucial signs were collected in our experiments for the endorsement of our view that the reprogrammed neurons are derived from astrocytes. Firstly, the designated AAV vectors driven by the GFAP promoter targets astrocytes specifically in WT mice (Fig. 4a, b and Supplementary Fig. S1e, f). Secondly, Cre-dependent expression of Neurog2 converts astrocytes successfully into neurons (Supplementary Fig. S3f, f′). Thirdly, we performed electrophysiological recording of Neurog2-iNs at 3DPI (data not shown) and 10DPI (Supplementary Fig. S2), and found that these induced cells exhibited astrocyte and immature neurons properties, respectively. These results suggest that Neurog2-iNs are derived from astrocytes.

Based on the data collected from WT and transgenic mice, we draw a conclusion that the majority of Neurog2-induced cells are glutamatergic neurons in the midbrain. Both double staining of in situ hybridization and immunostaining on brain sections of viral-injected WT mice manifested that most of the Neurog2/mCherry-positive cells are colabeled with VGLUT2, but not with Gad1. AAV-mCherry virus-infected cells maintain the identity of the astrocyte (Fig. 3a–h, u). Meanwhile, AAV-Neurog2/mCherry virus-induced neuronal cells colabeled with GFP in VGLUT2-GFP transgenic mice (Fig. 3i–p, v). From a rigorous point of view, a test for the control vector of AAV-mCherry in VGLUT2-GFP and Gad1-GFP transgenic mice should be performed to strengthen the conclusion. In the spinal cord, Neurog2-induced cells comprised both the excitatory cells and inhibitory cells (Fig. 4i, j).

Neurog2-iN cells would develop into electrophysiologically functional NeuN^+^ cells (Figs. 2e, f and 5f, d) in vivo and form synapses with local neurons (Fig. 2k, l). Remarkably, iN cells located in the spinal cord receive inputs from peripheral stimuli (Fig. 5j–m), our work firstly demonstrates the functional integration of reprogrammed neurons into local circuits in the spinal cord in vivo. Reactive astrocytes that exhibit substantial heterogeneity at multiple levels are generated in response to chronic neurodegenerative disease, or acute trauma^43^. The conversion from excessive astrocytes to neurons could be considered as a hopeful therapeutic approach^5,44^. Strikingly, the conversion from astrocytes to neurons induced by Neurog2 was spotted in the lesioned part of the spinal cord (Fig. 6f–h). Unlike the Sox2-mediated indirect conversion of astrocytes into neurons^44^, Neurog2-mediated reprogramming has a better performance in efficiency and neural maturation. Thus, our findings provide committed insights for the future application of Neurog2-iN cells in the reconstruction of damaged neural circuits.

## Supplementary information

Supplementary Figure Legends.

Supplementary Figure 1.

Supplementary Figure 2.

Supplementary Figure 3.

## Data Availability

All data generated or analyzed during this study are included in this published article.
