# Supplementary material for: Neurog2 directly converts astrocytes into functional neurons in midbrain and spinal cord
Source: Cell Death Dis. 2021 Mar 1;12(3):225. doi: 10.1038/s41419-021-03498-x (PMC7921562; doi:10.1038/s41419-021-03498-x)
Supplement: Supplementary file 1 — Supplementary Figure Legends. [file 41419_2021_3498_MOESM1_ESM.docx]

**Supplementary Figure Legends**

**Supplementary Figure 1. GFAP-AAV vectors target astrocytes of the dorsal midbrain in vivo.**

(**a-d**) Double staining of mCherry (**a**) and Neurog2 (**b**) in dorsal midbrain infected with AAV-Neurog2/mCherry at 3 DPI, the nucleus was stained by DAPI (**c**). Arrows in merged image (**d**) indicates colabeled cells. (**e-h'**) Double staining of mCherry and S100β on sections of the dorsal midbrain from WT mice infected with AAV-mCherry (**e**,**e'**,**g**,**g'**) or AAV-Neurog2/mCherry (**f**,**f'**,**h**,**h'**) at 3 DPI or at 30 DPI. **e',f',g', h'** are higher magnification of the boxed areas in **e**,**f**,**g**,**h**, respectively. Arrows indicatemCherry colabeled with S100β (**e'**,**f'**,**g'**) and arrowheads indicate mCherry non-labeled with S100β (**h'**). (**i**) Double staining images of mCherry and S100β on sections of the dorsal midbrain infected with AAV-Neurog/mCherry (left) or AAV-mCherry (right) at 30 DPI. Adjacent regions refer to an area close to the iNs region (AAV-Neurog2/mCherry infected range) without any visible mCherry. AAV-mCherry regions refer to the AAV-mCherry infected area. The three regions were randomly chosen and have a 450 x 500 pixels. (**j**) The statistical data of the average S100β^+^ cell numbers per region showed in (**i**). A one-way ANOVA revealed no significant effect of group (p > 0.05), followed by a Tukey’s Multiple Comparison test. "n.s." represents p > 0.05; (**k**) Double staining of mCherry and Neurog2 of dorsal midbrain infected with AAV-Neurog2/mCherry at 30 DPI, Arrows indicate the colabeled cells. Scale bars, 25 μm. (**l**,**m**) The comparison of membrane capacitance (**l**) and input resistance (**m**) among AAV-mCherry infected cells, local neurons and AAV-Neurog2/mCherry infected cells. A total of 31 cells, 14 cells, and 15 cells were recorded in astrocytes, local neurons, and Neurog2-iNs, respectively. “**”, and “***” represents 0.001 < p < 0.01, and p < 0.001, respectively; n.s. refers as nonsense.

**Supplementary Figure 2. The electrophysiological properties of Neurog2-iNs at 10 DPI and 30 DPI in midbrain.**

(**a-c**) Three typical responses to injected voltage step of Neurog2-iNs at 10 DPI. (**d-e**) Percentages of induced cells with three different degrees of membrane excitability (non-active, sAP, and mAP) at 10DPI (n = 7) (**d**) and 30DPI (n = 15) (**e**) after infection with the AAV–Neurog2/mCherry viruses. (**f**) Quantitative analysis of the AP amplitude of the cells infected with the AAV–Neurog2/mCherry viruses at 10 DPI, 30 DPI, and native local neurons. A one-way ANOVA revealed a significant effect of group (F[2,33] = 4.238, p = 0.02), followed by a Tukey’s Multiple Comparison test. 0.01< * p < 0.05.

**Supplementary Figure 3.** **Neurog2-iN cells do not pass through a proliferative state.** (**a-b'**) Double staining of GFP (green) and S100β (red) on sections of the dorsal midbrain from adult Aldh1l1-Cre mice at 3 DPI after infection of the control virus AAV-FLEX-NLSGFP (**a**,**a'**) or AAV-FLEX-Neurog2/GFP (,**b**,**b'**). **a'**, **b'** are higher magnifications of the boxed areas in **a**, **b**, respectively. Arrows in (**a',b'**) showed that GFP colocalized with S100β. **c-f'** Double staining of GFP (green) and NeuN (red) on sections of the dorsal midbrain from Aldh1l1-Cre mice at 3 DPI or 30 DPI after infection of the control virus AAV-FLEX-NLSGFP (**c**,**c'**,**d**,**d'**) or AAV-FLEX-Neurog2/GFP (**e**,**e'**,**f**,**f'**). **c'**, **d'**, **e'**,**f'** are higher magnifications of the boxed areas in **c**, **d**, **e**, **f**, respectively. Arrows in (**f'**) showed that GFP colocalized with NeuN. Arrowheads indicate GFP^+^NeuN^-^ cells (**c'**,**d'**,**e'**). **g-h''''** AAV-CAG-FLEX-Ngn2-GFP and AAV-hSyn-DIO-ChrimsonR-P2a-tdTomato viruses were injected into the midbrain of Aldh1l1-cre mice. (**g**)Double staining of mCherry and GFP on sections infected with virus at 30 DPI. (**h-h''''**) Double staining of mCherry,GFP, and c-Fos on sections activated with laser at 30 DPI. (**i**,**j**) Double staining of mCherry and BrdU on sections of dorsal midbrain from WT mice infected with the control virus AAV-mCherry (**i**) or virus AAV-Neurog2/mCherry (**j**) at 30 DPI. mCherry was not colocalized with BrdU (arrowheads). Upper left, upper right, bottom left, and bottom right represent mCherry, BrdU, DAPI and merged images, respectively. Scale bars, 50 μm.
