# Supplementary figures and images for: Neurog2 directly converts astrocytes into functional neurons in midbrain and spinal cord
Source: Cell Death Dis. 2021 Mar 1;12(3):225. doi: 10.1038/s41419-021-03498-x (PMC7921562; doi:10.1038/s41419-021-03498-x)

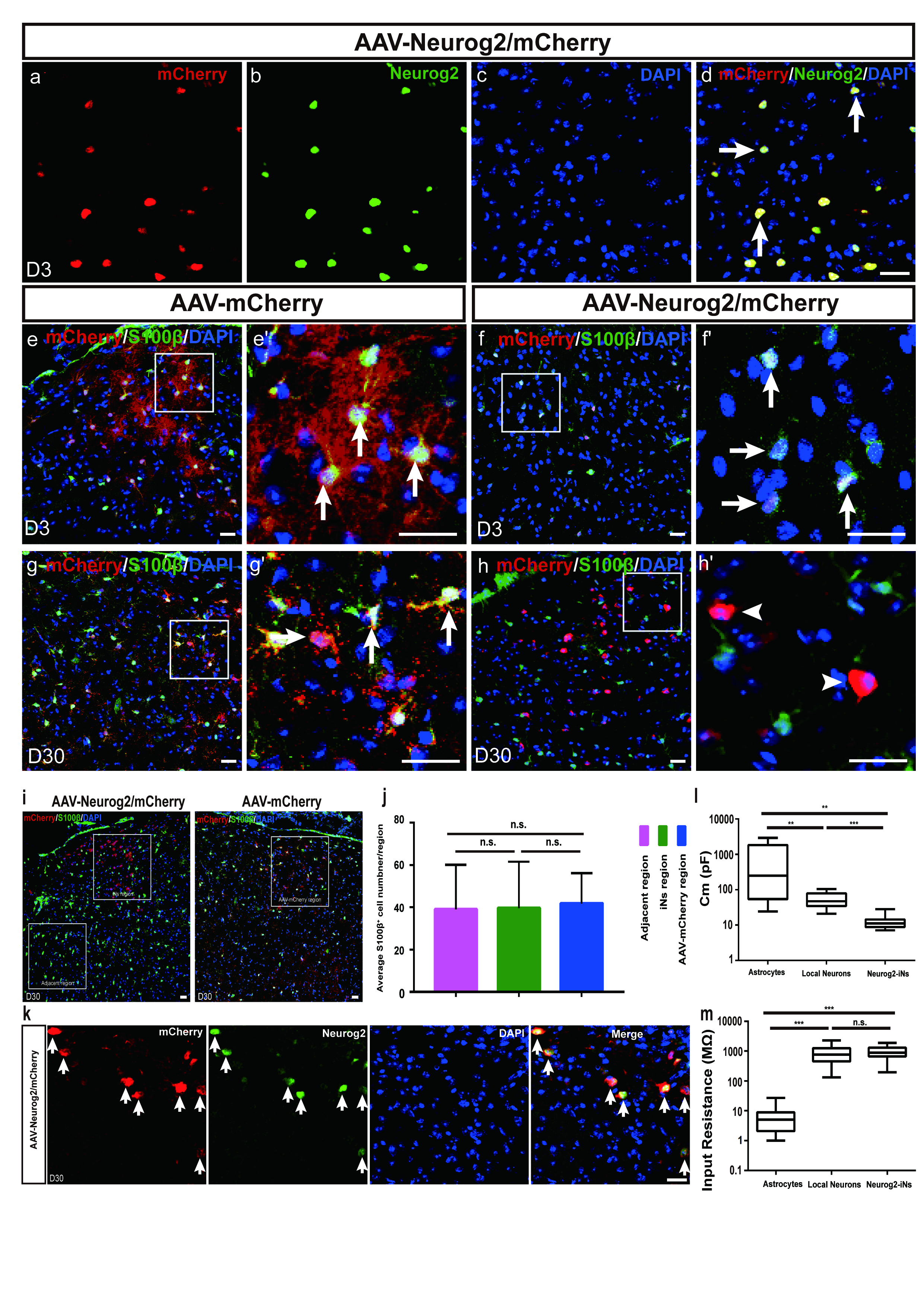

Supplement: Supplementary file 2 — Supplementary Figure 1. [file 41419_2021_3498_MOESM2_ESM.tif]

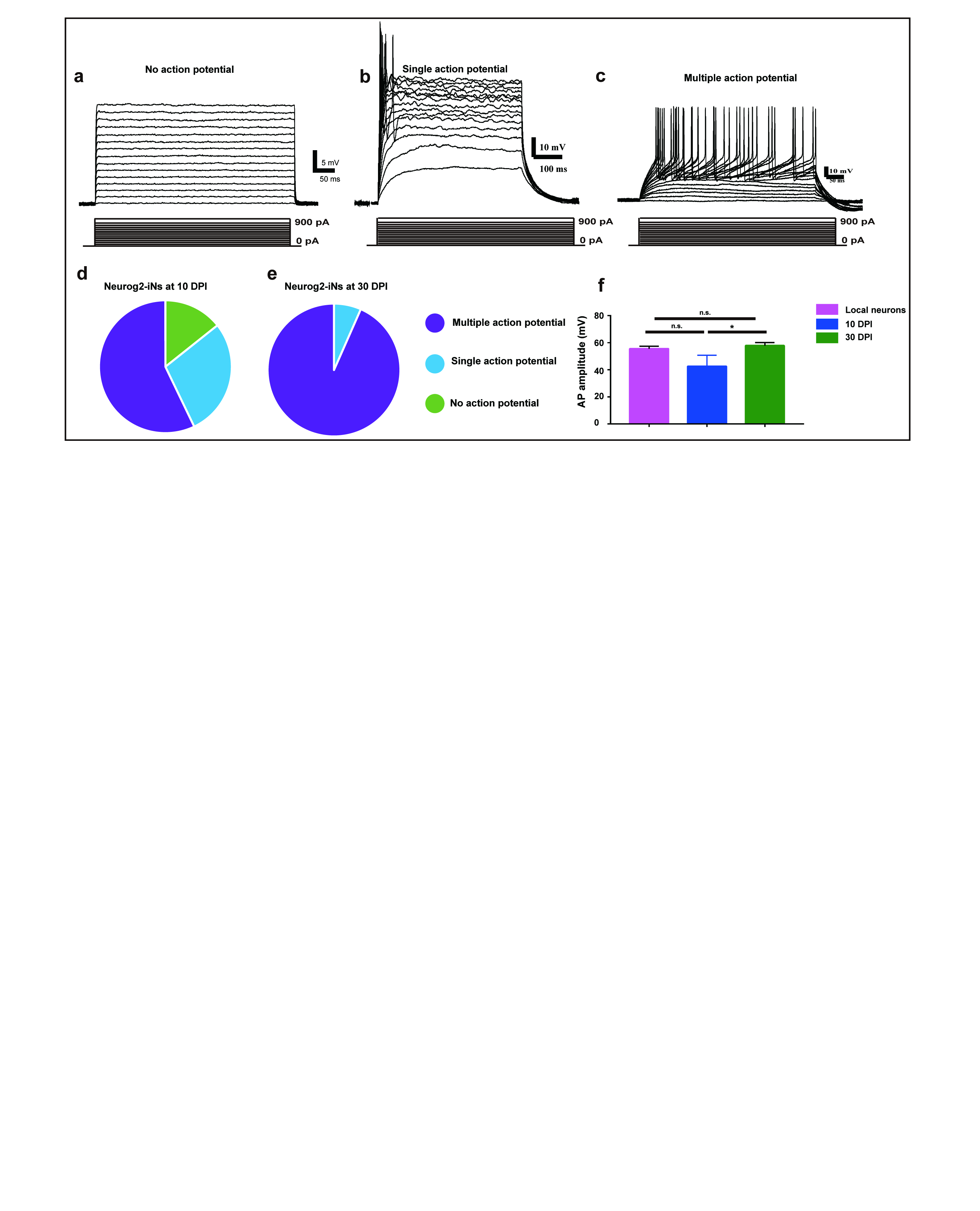

Supplement: Supplementary file 3 — Supplementary Figure 2. [file 41419_2021_3498_MOESM3_ESM.tif]

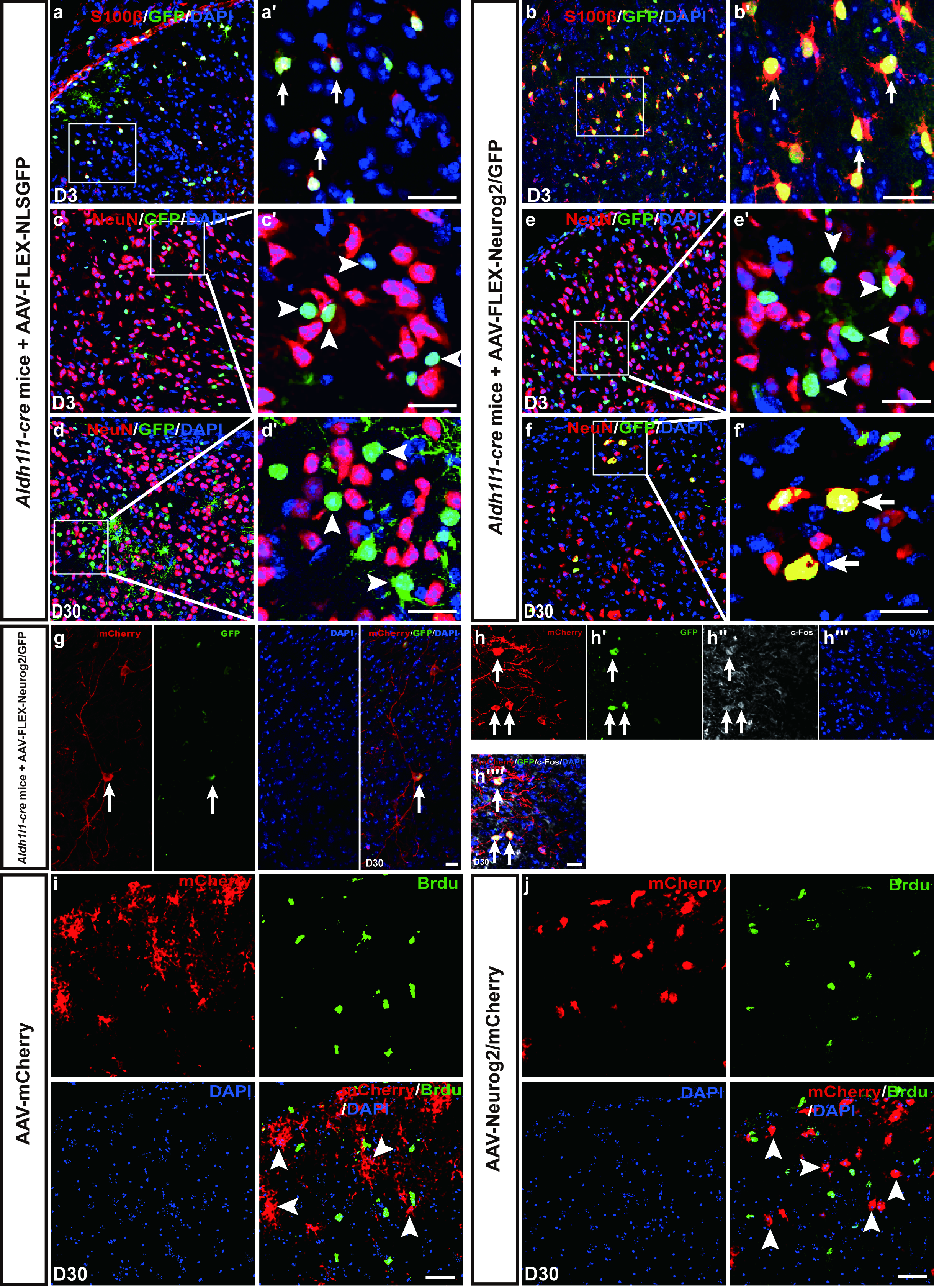

Supplement: Supplementary file 4 — Supplementary Figure 3. [file 41419_2021_3498_MOESM4_ESM.tif]
